# Quantitative Estimation of Naproxen in Tablets Using Ibuprofen Sodium as Hydrotropic Agent

**DOI:** 10.4103/0250-474X.56020

**Published:** 2009

**Authors:** R. K. Maheshwari, G. Wanare, Nitika Chahar, Prajakta Joshi, Nishi Nayak

**Affiliations:** Department of Pharmacy, S. G. S. I. T. S., 23, Park Road, Indore-452 003, India

**Keywords:** Hydrotropy, ibuprofen sodium, naproxen titrimetry

## Abstract

In the present investigation 0.5 M ibuprofen sodium solution has been used as hydrotropic solubilizing agent for naproxen, a poorly water-soluble drug and in it there was more than 350 fold enhancement in the solubility of naproxen as compared to the solubility in distilled water. Therefore, this hydrotropic solution was employed to extract out the drug from its tablet dosage form for quantitative estimation by titrimetry. The naproxen has been successfully analyzed in tablets. The results of analysis obtained by proposed method compared well with those by corresponding British pharmacopoeial method involving the use of methanol. The proposed method was also validated by recovery studies. Presence of ibuprofen sodium and common excipients did not interfere in analysis. Proposed method is new, simple, economic, safe, rapid, accurate, reproducible and environment-friendly.

Use of hydrotropic solubilizing agent is one of the several methods reported in the literature to enhance solubility of poorly water-soluble drugs. Hydrotropy is a solubilization phenomenon whereby addition of a large amount of a solute results in an increase in aqueous solubility of another solute. Concentrated aqueous solutions of large number of hydrotropic agents like sodium salicylate, sodium benzoate, urea, sodium acetate, sodium ascorbate, niacinamide and sodium citrate have been employed to enhance aqueous solubility of poorly water-soluble drugs[1–20]. By applying hydrotropic solubilization phenomenon, a large number of poorly water-soluble drugs have been quantitatively estimated by Maheshwari and his associates[1–16]. The main objective of the present research is to explore the possibility of employing a new inexpensive hydrotropic agent, ibuprofen sodium to solubilize a poorly water-soluble drug, naproxen for its titrimetric analysis precluding the use of an organic solvent.

Various organic solvents are used for solubilization of many of the poorly water-soluble drugs to facilitate their titrations (acid-base) and spectrophotometric estimations. Most of these solvents are costlier, toxic, environment pollutants. The above account prompted us to investigate a new method employing 0.5 M ibuprofen sodium solution as a hydrotropic solubilizing agent in place of organic solvent for solubilization purpose to facilitate the titrimetric analysis rather than official spectrophotometric (BP) method[21] involving use of organic solvent for estimation of poorly water-soluble NSAID, naproxen [(RS) 6 methoxy-2-naphthalene acetic acid] in tablets with sodium hydroxide solution as titrant.

A Shimadzu UV/Vis recording spectrophotometer (model-UV-160 A) with 1 cm matched silica cells was employed for spectrophotometric analysis. The bulk drug sample of naproxen was generously supplied by M/s Alkem Laboratories Ltd., Mumbai, India. Commercial tablets of naproxen (Naprosyn, RPG life Sciences Ltd. and Xenar-CR, Elder Health Care Ltd.) were procured from the local Pharmacy. Other chemicals and solvents were of analytical grade. In order to prepare 0.5 M ibuprofen sodium solution, 10 g sodium hydroxide was dissolved in 200 ml distilled water and 51.6 g ibuprofen bulk drug was added little at a time with shaking. After complete addition, the pH was maintained to 7.5-8.0 with sodium hydroxide solution and volume was made up to 250 ml with distilled water.

In preliminary solubility studies of naproxen, it was found that there was more than 350 times enhancement in the solubility in 0.5 M ibuprofen sodium solution as compared to water-solubility at room temperature. Thus, it was thought worthwhile to employ 0.5 M ibuprofen sodium solution in the estimation of naproxen tablets.

In analysis of naproxen tablets by official method of BP 2002[21], twenty tablets of naproxen were weighed and powdered. Tablet powder equivalent to 50 mg of naproxen was shaken with 70 ml of methanol for 30 min and sufficient methanol was added to produce 100 ml. After filtration, 10 ml filtrate was diluted to 50 ml with methanol. Absorbance of this solution was measured at 331 nm using spectrophotometer. From the absorbance obtained by repeating the operation using a 0.01% w/v solution of naproxen drug sample in methanol and the drug content was calculated (Table 1). Each analysis was done for formulation I and II in triplicate.

**TABLE 1:** RESULTS OF ANALYSIS OF NAPROXEN TABLET FORMULATIONS

Amount of drug present in tablet powder analyzed by proposed method (mg)	Amount of drug present in tablet powder analyzed by BP method (mg)	Amount of drug found (mg)				Percent estimated			
	
		Formulation-I		Formulation-II		Formulation-I		Formulation-II	
			
		PM	BPM	PM	BPM	PM	BPM	PM	BPM
400	50	400.83	48.86	393.71	49.22	100.21	97.72	98.43	98.44
400	50	392.80	51.02	393.01	49.57	98.20	102.04	98.25	99.14
400	50	394.73	49.22	396.55	50.13	98.68	98.44	99.14	100.26

PM stands for the proposed method, BPM is the British Pharmacopoeial method. Formulation I is Naprosyn of RPG life Sciences Ltd and Formulation II is Xenar-CR of Elder Health Care Ltd.

By the proposed method of analysis tablet powder equivalent to 400 mg naproxen was accurately weighed and transferred to a conical flask. After adding 100 ml of 0.5 M ibuprofen sodium solution, the flask was shaken for 10 min for solubilization of the drug. Titration was performed using 0.1 M sodium hydroxide solution using phenolphthalein solution as indicator. Blank determination was carried out and necessary correction was made to calculate the drug content (Table 1).

By performing the solubility studies, it was found that enhancement in aqueous solubility of naproxen in 0.5 M ibuprofen sodium solution was more than 350 fold as compared to its solubility in distilled water. There was negligible enhancing effect on solubility of this drug in buffer of pH 8.2 and such enhancement in solubility of this drug in 0.5 M ibuprofen sodium solution could therefore be attributed to hydrotropic solubilization phenomenon and not due to change in pH. The mean percent estimations (Table 2) for the tablet formulations were 99.03 and 98.61 in case of the proposed method. Since these values are very close to 100, this proves the accuracy of the proposed method. These values also compare very well with the values of the mean percent estimations, 99.40 and 99.28 (Table 2) obtained in case of the pharmacopoeial method which is a standard method. The validation of the proposed method is further confirmed by the low values of statistical parameters viz. standard deviation, percent coefficient of variation and standard error (Table 2). The mean percent recovery values obtained by use of the proposed method ranged from 98.77 to 101.38. Again, these values are close to 100 and the values of statistical parameters viz. standard deviation (ranged between 0.624 to 1.930), percent coefficient of variation (ranged between 0.625 to 1.954) and standard error (ranged between 0.360 to 1.114) are satisfactorily low which further confirm the validity of the proposed method. However in the proposed method, 0.5 M ibuprofen sodium solution has been used as a hydrotropic solubilizing agent for solubilization of drug to carryout titration with sodium hydroxide solution. In the analysis of naproxen tablets by the proposed method, no sophisticated instrument was required and also there was no involvement of any organic solvent. Thus, it may be concluded that the proposed method of analysis is new, simple, accurate, precise, safe, eco-friendly and cost-effective. Definitely, there is further scope of hydrotropic agent, ibuprofen sodium solution as solubilizing agent for other poorly water-soluble drugs to facilitate titrimetric analysis precluding the need of organic solvents which are mostly toxic, costlier and pollutant.

**TABLE 2:** STATISTICAL EVALUATION OF ANALYSIS OF TABLETS

Tablet formulation	Mean % estimation		Standard error		%Coefficient of variation		Standard error	
				
	PM	BPM	PM	BPM	PM	BPM	PM	BPM
I	99.03	99.40	1.050	2.314	1.060	2.328	0.606	1.336
II	98.61	99.28	0.470	0.918	0.477	0.925	0.271	0.530

PM stands for the proposed method, BPM is the British Pharmacopoeial method. Formulation I is Naprosyn of RPG life Sciences Ltd and Formulation II is Xenar-CR of Elder Health Care Ltd.
